# Adoptive transfer of bone marrow-derived dendritic cells (BMDCs) alleviates OVA-induced allergic airway inflammation in asthmatic mice

**DOI:** 10.1038/s41598-020-70467-3

**Published:** 2020-08-17

**Authors:** Kan Xu, Nan Wu, Zhihui Min, Zheng Li, Tao Zhu, Chunfang Liu, Yuzhen Zeng, Juan Song, Ruolin Mao, Hong Ji, Zhilong Jiang, Zhihong Chen

**Affiliations:** 1 Geriatric Department of Zhongshan Hospital, Shanghai Institute of Respiratory Disease, Fudan University, Shanghai, China; 2 Respiratory Division of Zhongshan Hospital, Shanghai Institute of Respiratory Disease, Fudan University, No. 180 Fenglin Road, Shanghai, China; 3 Research Center of Zhongshan Hospital, Fudan University, Shanghai, China; 4 Department of Respiratory Medicine, Second Affiliated Hospital of Chongqing Medical University, Chongqing, China; 5 Department of Anatomy, Physiology and Cell Biology, School of Veterinary Medicine, University of California, Davis, CA USA; 6 California National Primate Research Center, Davis, CA USA; 7 Department of Laboratory Medicine, Huashan Hospital, Shanghai Medical College, Fudan University, Shanghai, China

**Keywords:** Asthma, Asthma, Therapeutics, Therapeutics

## Abstract

Airway dendritic cells (DCs) are recognized as important factors in the mechanisms of allergic inflammatory diseases. Suppressor of cytokine signaling 3 (SOCS3) is involved in regulating the functions of T cells and macrophages, but the roles of SOCS3-expressing DCs in the pathogeneses of allergic inflammatory diseases are still controversial. We compared the effects of adoptively transferred SOCS3^−/−^ and SOCS3^+/+^ bone marrow-derived DCs (BMDCs) on airway inflammation in ovalbumin (OVA)-sensitized asthmatic mice. Adoptive transfer of mature DCs (lipopolysaccharide [LPS]-induced DCs, DClps) with or without SOCS3 gene expression significantly ameliorated allergic airway inflammation. SOCS3^−/−^ DCs slightly attenuated BMDC-induced immunogenic tolerance. DClps migrated to OVA-sensitized lungs with higher efficiency than immature DCs (DCim). DClps with or without SOCS3 greatly improved lung pathology scores and alleviated airway inflammatory cell infiltration after adoptive transfer into mice; they also increased interleukin-10 (IL-10) and transforming growth factor-β (TGF-β) production and inhibited signal transducer and activator of transcription (STAT) 4 and STAT6 signaling in the lungs after OVA sensitization. In conclusion, the BMDC adoptive transfer-induced immunogenic tolerance in OVA-sensitized mice might not be due to SOCS3 gene depletion. BMDC adoptive transfer may be developed into a new approach that alleviates asthma by modulating the balance between immune tolerance and inflammation.

## Introduction

Airway dendritic cells (DCs) play crucial roles in initiating effective adaptive immune responses against invading pathogens and inducing immune tolerance toward innocuous inhaled antigens. Exploiting the tolerogenic function of DCs might be a novel way to treat allergic airway diseases. However, deletion of DCs in the lungs is infeasible, as indicated by studies in which DC−/− mice have been found to exhibit severe viral respiratory infections and systematic illness^1^. Fine-tuning the balance between tolerogenic and immunogenic lung DCs is a major goal in anti-inflammation research. Emerging literature has demonstrated that different DC subsets and discrete functional states of DCs might be responsible for promoting tolerance to inhaled antigenic substances. For example, Nakagome et al. reported that interleukin (IL)-10-treated DCs decrease airway allergic inflammation in mice^2^. In addition, it has been shown that plasmacytoid DCs (pDCs) play an important role in inhalation tolerance. Mice in which pDCs are specifically depleted develop the features of severe asthma after exposure to nebulized harmless antigens^3^. Steroids can modulate the functions of DCs in the lungs of patients with allergic asthma by activating indoleamine 2,3-dioxygenase (IDO) enzymes in DCs^4,5^. Furthermore, vitamin D3-incubated bone marrow-derived DCs (BMDCs) express relatively low levels of major histocompatibility complex class II (MHCII) and costimulatory molecules, which ultimately attenuates DC-T cell interactions and T cell activation^6^.

Suppressor of cytokine signaling 3 (SOCS3) is central in negatively regulating signal transducer and activator of transcription (STAT) 3, STAT4, STAT1 and STAT5 signaling after stimulation with IL-6, IL-11, IL-27, etc. Kubo et al. found that SOCS3 mRNA expression is increased in eosinophils and CD4+ T cells in asthma and nonasthmatic eosinophilic bronchitis. T cell-specific deletion of SOCS3 impairs the T helper (Th) 2 response and increases Th1 responses^7^. However, deletion of SOCS3 in hematopoietic cells results in severe inflammatory disease during adult life that is not rescued by IL-6 deletion^8^. In addition, SOCS3 gene knockdown in macrophages results in activation of STAT1 and induction of type I interferon (IFN) responses upon IL-6 stimulation^9^. Thus, the roles of the SOCS3 gene in DC functional states and the cognate interaction of SOCS3 with T cells have been controversial.

Herein, we critically assessed the effects of the SOCS3 gene in BMDCs on cell proliferation and activation by coculturing SOCS3^−/−^ BMDCs with CD4 T cells. Then, DCs with SOCS3 gene deletion in different functional states were adoptively transferred into ovalbumin (OVA)-sensitized mice, and lung pathological injury and airway inflammatory cell infiltration were evaluated. The underlying cellular and molecular mechanisms were also studied.

## Results

### SOCS3 deficiency increased the DC-induced proliferation and cytokine production of T lymphocytes

**Figure 1:**
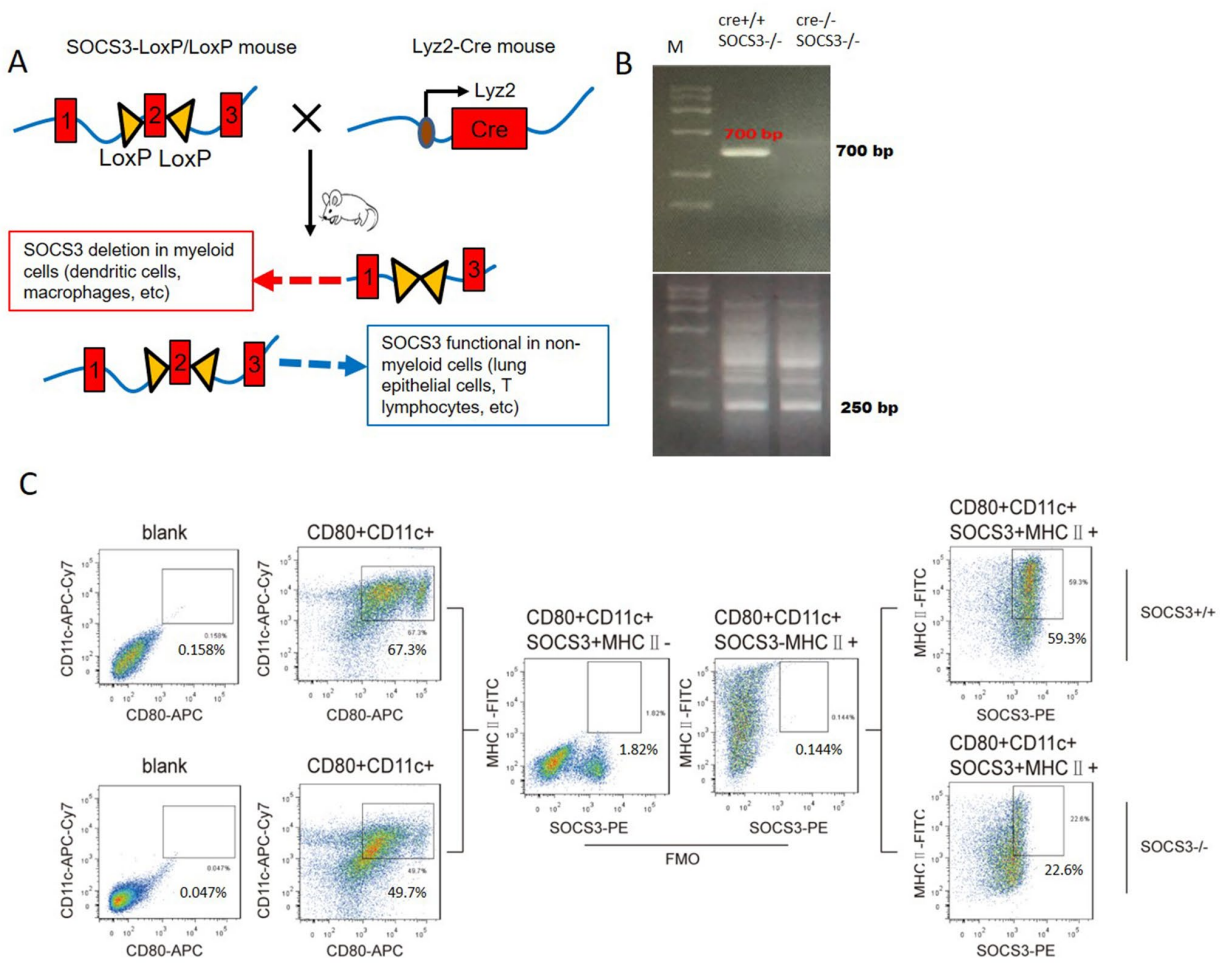
Generation of SOCS3(Lyz2cre) mice and identification of SOCS3^−/−^ BMDCs. (**A**) Schematic diagram of the generation of SOCS3(Lyz2cre) mice. Floxp-flanked SOCS3 mice were back-crossed with Lyz2-Cre transgenic mice to create SOCS3 knockout mice with SOCS3 conditional knockout in myeloid cells, such as DCs or macrophages. (**B**) The genotypes of SOCS3(Lyz2cre) mice identified by analyzing mouse tails by PCR. The Cre + loci were identified as 700 bp. The FloxP-flanked exon 2 null SOCS3 loci were identified as 250 bp. (**C**) Expression of SOCS3 in BMDCs evaluated by flow cytometry. BMDCs were gated on CD11c + CD80 + MHCII + cells. A PE-conjugated anti-SOCS3 antibody was used to detect SOCS3 protein expression. In SOCS3^−/−^ mice, SOCS3 protein expression was reduced by approximately 62.4%. One representative dot plot is shown (SOCS3 and MHCII expression was assessed by comparing with the corresponding fluorescence-minus-one (FMO) control).

To investigate the role of SOCS3 in airway inflammation, we created conditional SOCS3-knockout (KO) mice according to the protocol in a previous study^10^. Briefly, SOCS3fl/fl mice were bred with mice transgenically expressing Cre under the control of the lysozyme 2 (Lyz2) promoter. The offspring SOCS3(Lyz2cre) mice lacked exon 2 of the SOCS3 locus in myeloid cells; this exon was deleted under the control of the Lyz2 promoter (Fig. 1A). To identify BMDCs with SOCS3 deficiency, we screened bone marrow cells expressing CD11c, CD80, and MHCII from each group and differentiated them into BMDCs in culture. Fluorescence-activated cell sorting (FACS) analysis showed that SOCS3 protein expression was significantly lower (62% lower) in SOCS3(Lyz2cre) mouse-derived BMDCs than in wild-type (WT) mouse-derived BMDCs (Fig. 1C). Western blot analysis confirmed that the expression of SOCS3 was decreased by 56% in SOCS3^−/−^ BMDCs (Supplementary Data 1).

**Figure 2:**
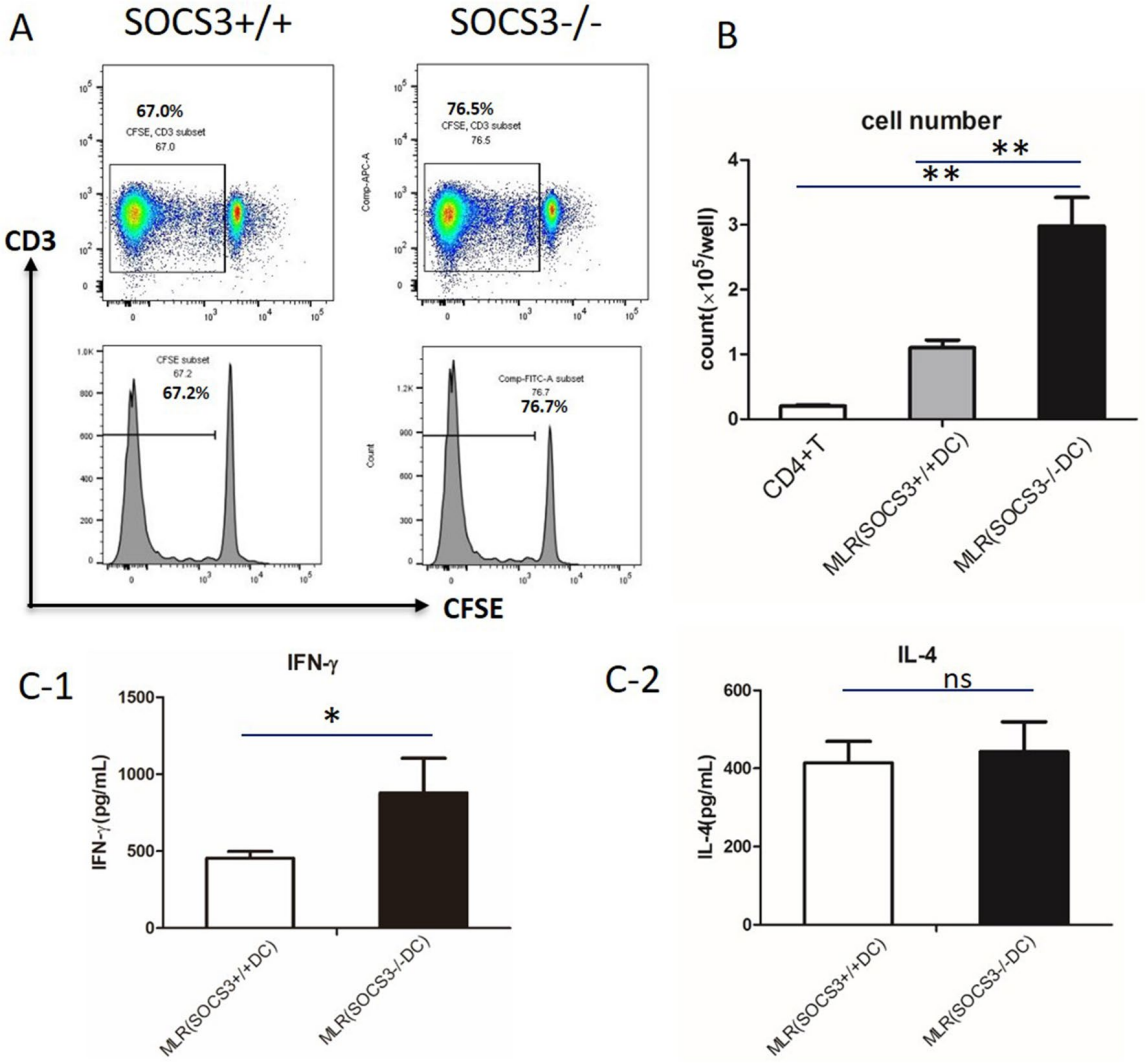
Mixed lymphocyte reaction (MLR) with SOCS3^−/−^ BMDCs. (**A**) Mitomycin-activated BMDCs cultured with CD4 + T cells (cell ratio of 1:4). T cell proliferation was assessed with CFSE staining. One representative plot is shown. (**B**) Number of CD4 + T cells after a 5-days MLR. (**C**) Cytokine release from CD4 + T cells after a 5-day MLR. (**C-1**) IFN-γ production; (**C-2**) IL-4 production. Data are representative of 3 independent experiments with similar results. The columns and error bars represent the mean and SEM (*P < 0.05, **P < 0.01, ns: no significant difference).

We performed an allogeneic mixed lymphocyte reaction (MLR) to evaluate whether a lack of SOCS3 in BMDCs affects interactions with T lymphocytes by assessing the function and proliferative ability of T lymphocytes. We distinguished T cells by staining cells in MLR culture with an anti-CD3 antibody. Carboxyfluorescein diacetate succinimidyl ester (CFSE) analysis and differential cell counting both demonstrated that SOCS3^−/−^ DCs induced more robust T cell proliferation than SOCS3^+/+^DCs (Fig. 2A,B). Surprisingly, SOCS3 deficiency increased the expression of IFN-γ by T cells (Fig. 2C1), but it did not influence the expression of IL-4 (Fig. 2C2).

### Lack of SOCS3 did not alter the therapeutic effect of DCs on allergic airway inflammation

**Figure 3:**
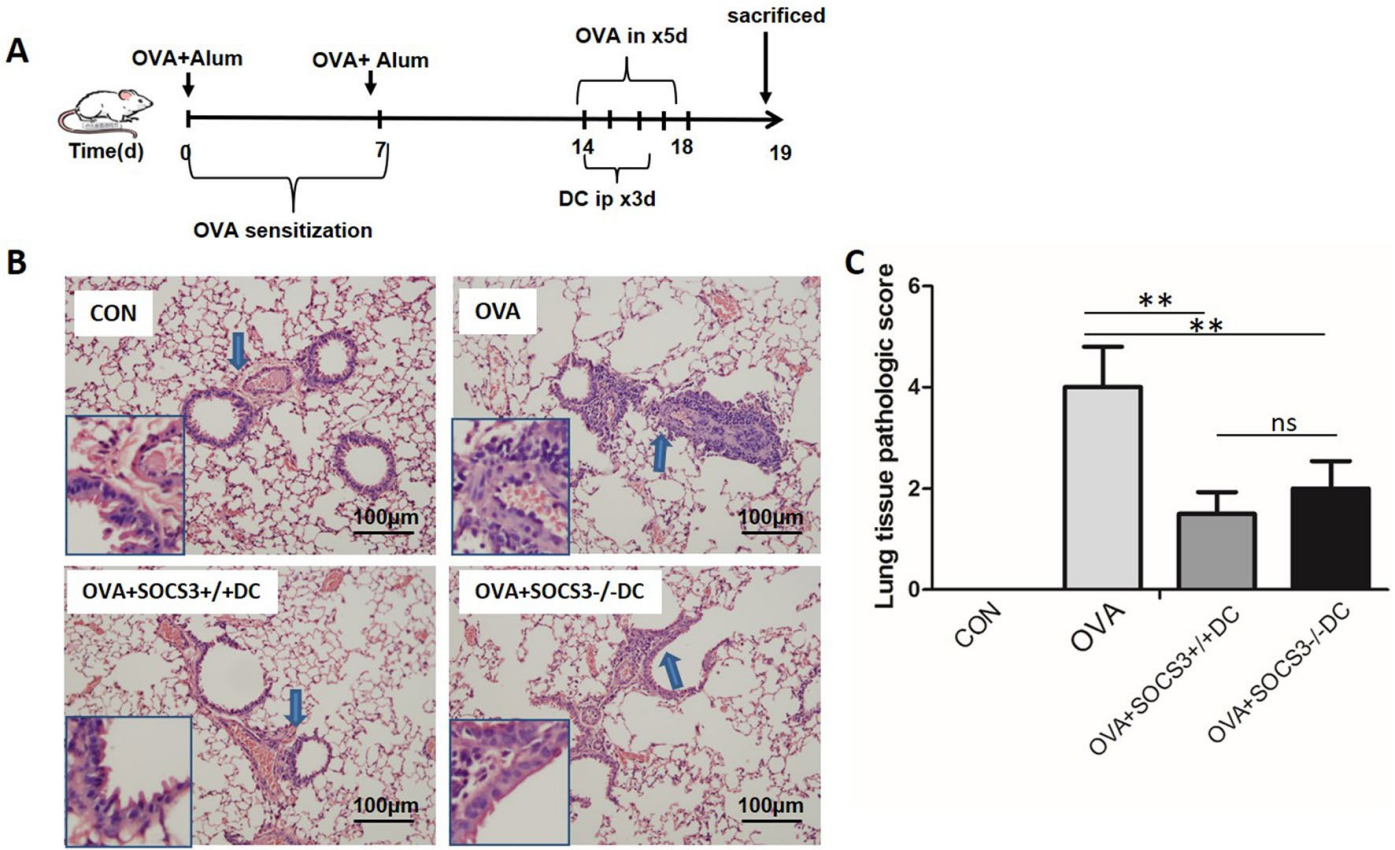
Effect of adoptively transferred BMDCs with or without the SOCS3 gene on lung pathology in OVA-induced asthmatic mice. (**A**) Protocol for OVA-mediated induction of allergic asthma and SOCS3^+/+^ DC or SOCS3^−/−^ DC adoptive transfer into OVA-sensitized mice via intraperitoneal injection (DCs pulsed with LPS before transfer). (**B**) Representative photomicrographs of lung sections stained with H&E and examined at × 100 magnification. The same experiment was repeated 3 times with similar results (n = 6 in each group). C. The scores for lung tissue pathology. The columns and error bars represent the mean and SEM (**P < 0.01, ns: no significant difference).

To further evaluate the effects of SOCS3 deletion, we generated an OVA-induced asthma mouse model and adoptively transferred DCs into the asthmatic mice (Fig. 3A). Transfer of DCs ameliorated lung tissue damage and decreased allergic airway inflammation; however, SOCS3 deficiency did not alter the therapeutic effect of DCs. In other words, DC therapy attenuated the inflammatory response in the lungs regardless of whether the mature DCs (lipopolysaccharide [LPS]-induced DCs, DClps) used for treatment expressed the SOCS3 gene (Fig. 3B,C). Notably, SOCS3^−/−^ DC therapy seemed to have a smaller beneficial effect on lung tissue pathological scores than SOCS3^+/+^DC therapy. However, the difference was not statistically significant (Fig. 3C).

**Figure 4:**
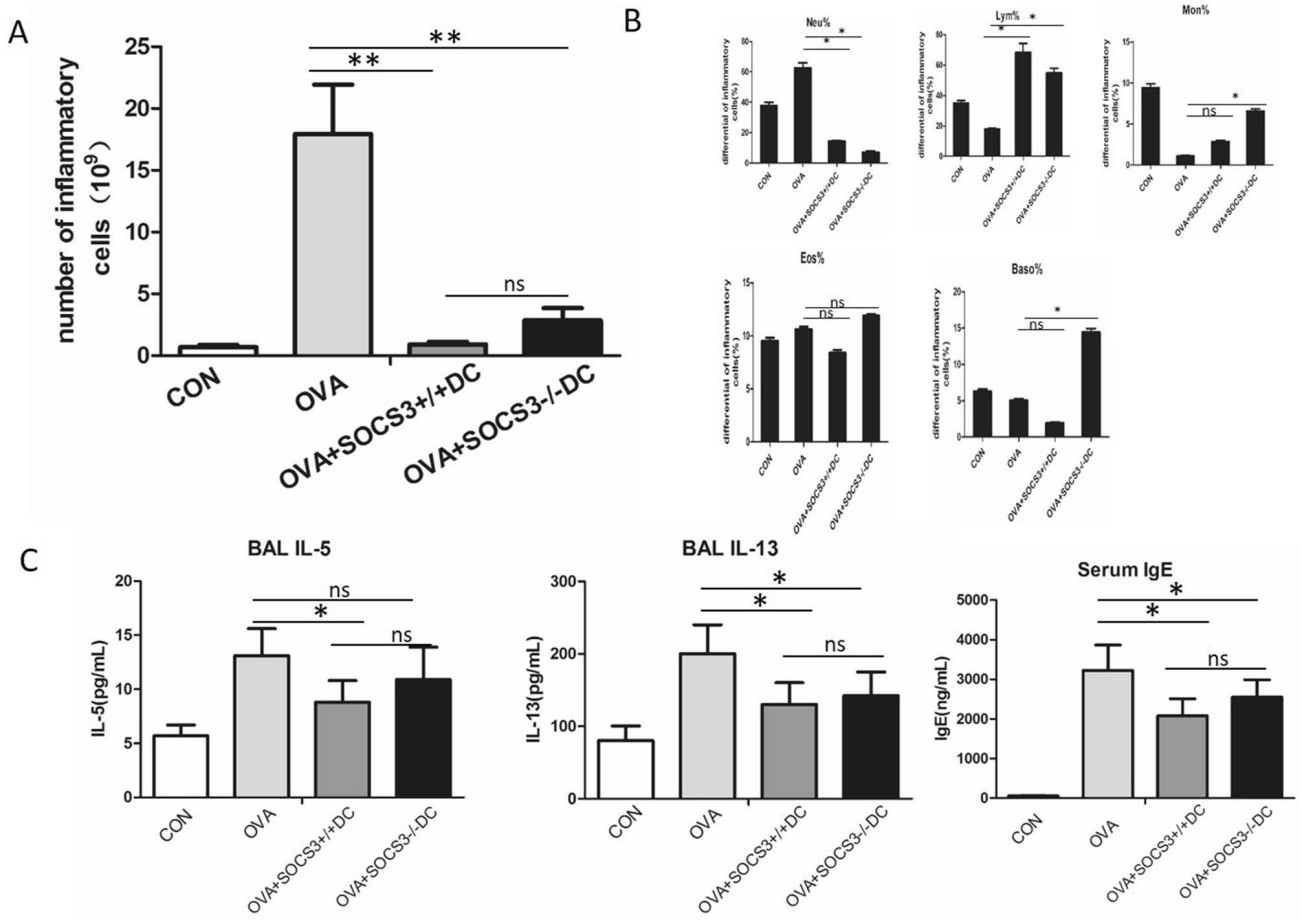
Adoptively transferred BMDCs with or without the SOCS3 gene alleviated allergic airway inflammation. (**A**) The total cell number in the bronchoalveolar lavage fluid (BALF). (**B**) The differential cell counts in the BALF. (**C**) The concentrations of Th2 cytokines in the BALF and serum IgE measured by ELISA. Data are representative of 3 independent experiments with similar results. The columns and error bars represent the mean and SEM (**P < 0.01, ns: no significant difference).

In addition, transfer of DCs reduced the total number of pulmonary inflammatory cells in the bronchoalveolar lavage (BAL) fluid (BALF) (Fig. 4A). An obvious reduction in neutrophil count and increase in lymphocyte count were observed. There was no change in eosinophil count after DC transfer (Fig. 4B). Correspondingly, the expression of IL-13 and immunoglobulin (Ig) E, which are related to the asthmatic Th2 response, was diminished after treatment with DCs (Fig. 4C). Unexpectedly, the inhibitory effect of BMDC adoptive transfer was not related to SOCS3 gene status (Fig. 4C).

### Proinflammatory and chemotactic effects of DClps and immature DCs (DCim)

In the above experiment, we demonstrated that transfer of DCs greatly ameliorated lung tissue damage and decreased allergic airway inflammation. Next, we cultured DCs in vitro to further elucidate whether the therapeutic effect of DCs was dependent on DC maturation.

**Figure 5:**
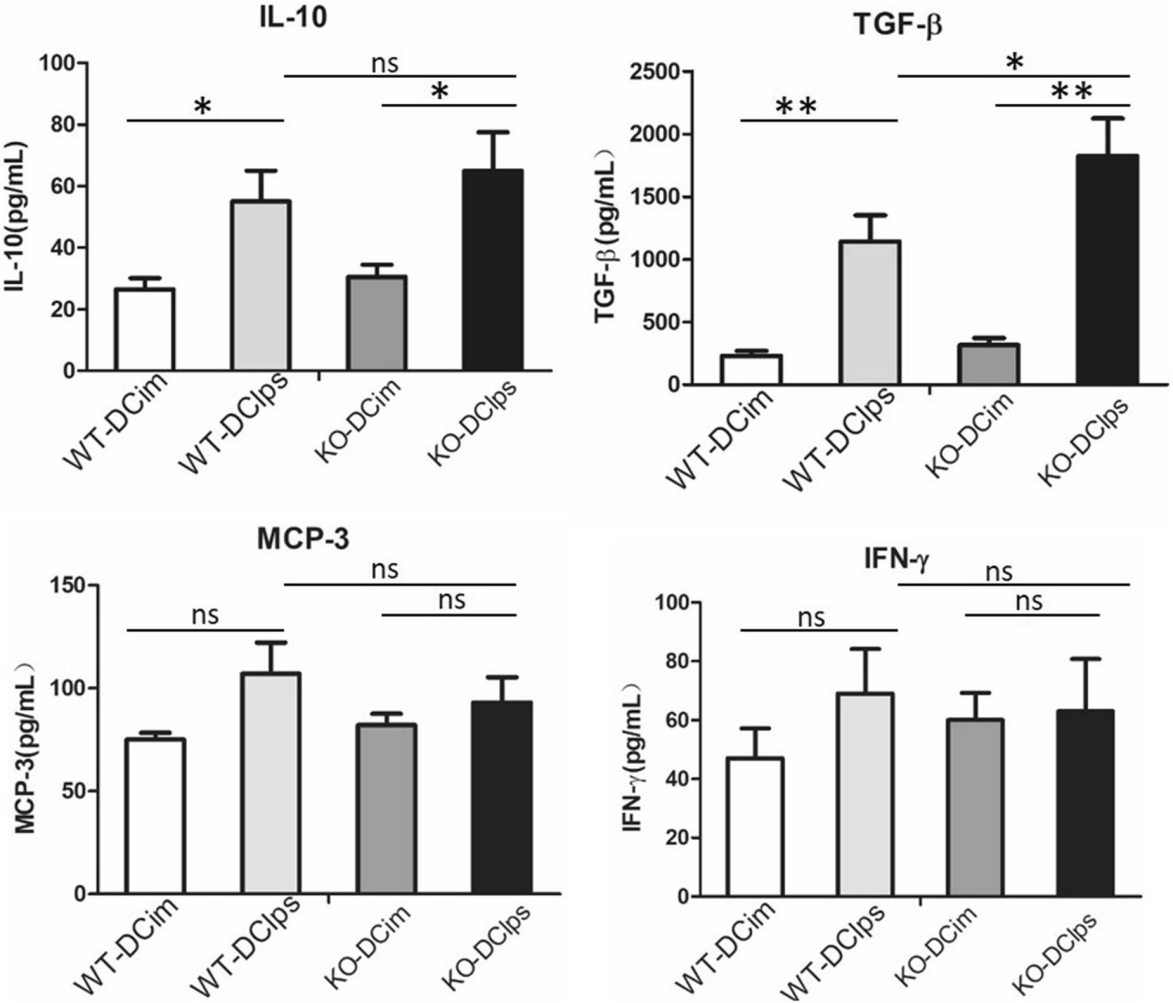
Activity of mature and immature DCs with or without the SOCS3 gene. BMDCs were generated from SOCS3^+/+^ or SOCS3^−/−^ mice and incubated with or without LPS (the cells incubated with LPS were considered mature DCs). The cells were then pulsed with OVA for 2 h, and the supernatant was collected for ELISA analysis. Data are representative of 3 independent experiments with similar results. The columns and error bars represent the mean and SEM (*P < 0.05, **P < 0.01, ns: no significant difference).

DCim were induced to differentiate into mature DCs in the presence of LPS (producing DClps) and were pulsed with OVA for 2 h. We then measured the levels of select related cytokines in DCim and DClps culture supernatants. ELISA revealed that the levels of IL-10 and transforming growth factor-β (TGF-β) were higher in DClps culture medium than in DCim culture medium and that the expression of MCP-3 and IFN-γ was unaffected by DC maturation (Fig. 5).

Among the DClps groups, SOCS3 deficiency had no effect on the expression of MCP-3 or IFN-γ. Surprisingly, the expression of TGF-β was mildly elevated in SOCS3^−/−^ DClps compared to SOCS3^+/+^DClps (Fig. 5).

### The maturity of DCs affects the efficiency of DC pulmonary migration following intraperitoneal injection

We attempted to track DCs that had been labeled with the fluorescent dye PKH26 to test whether DCs could migrate to the lungs successfully and to assess whether the efficiency of pulmonary migration was related to the maturation state of DCs.

**Figure 6:**
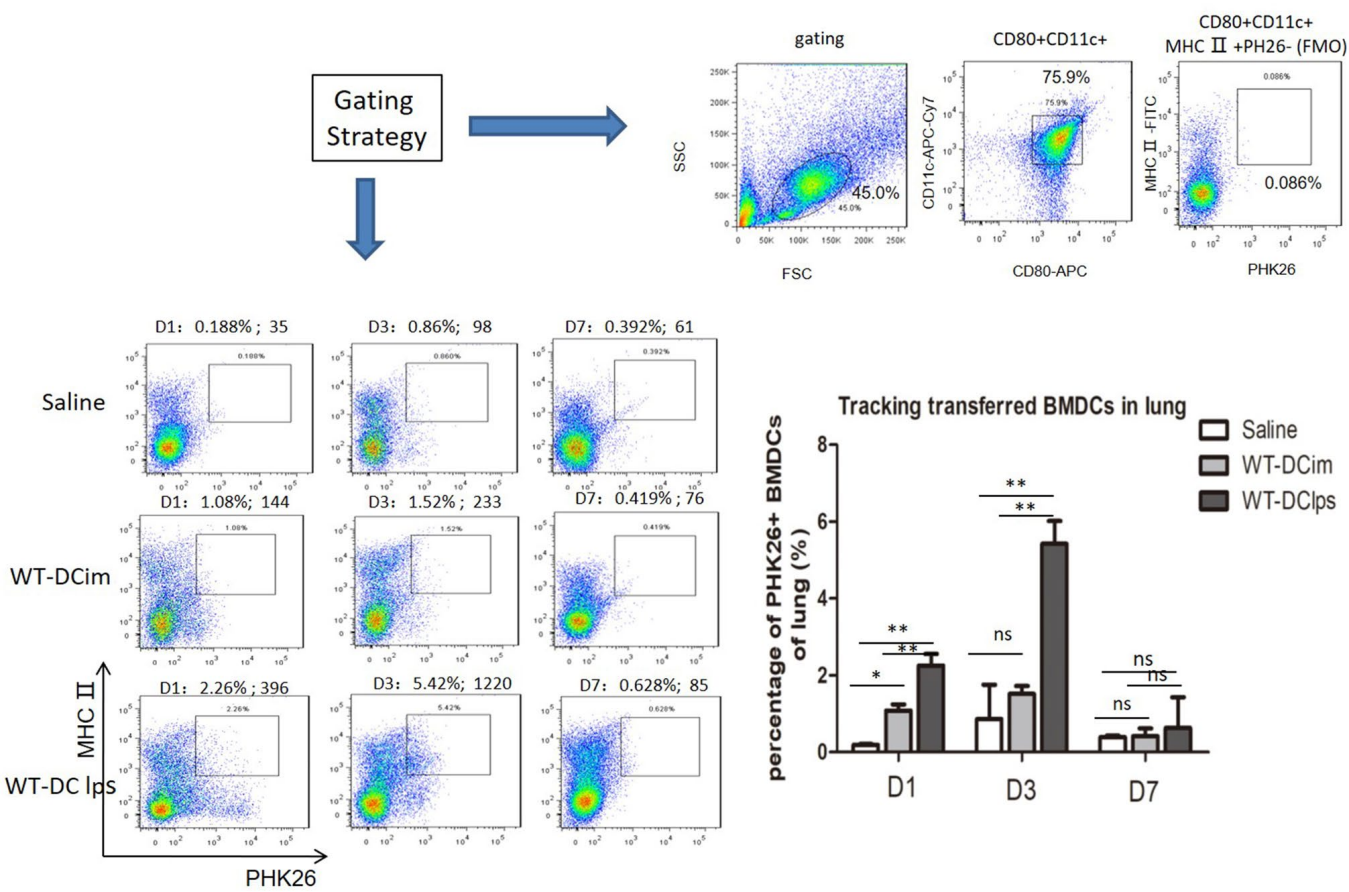
Tracking adoptively transferred wild-type BMDCs in OVA-induced asthmatic mice. Wild-type BMDCs pulsed with or without LPS were labeled with PKH26 (WT-DClps and WT-DCim) or saline-diluted PKH26 and transperitoneally transferred into OVA-sensitized mice. On day 1, 3, or 7, mice were sacrificed. BMDCs were screened by gating on CD11c + CD80 + MHCII + cells in the mononuclear cell population of the lungs. WT-DClps labeled with PKH26 were tracked and reached 5.42% of the BMDC population in the lungs (the absolute number of WT-DClps migrating to the lungs was 1,220), and this proportion was higher than that of WT-DCim and saline control on day 3. The gating strategy and the fluorescence-minus-one (FMO) control (PKH26 negative) are presented. Histogram represents the percentage of PHK26 + BMDCs adoptively transferred into the lung within a week. Data are representative of 3 independent experiments, and three mice were used in each group. The columns and error bars represent the mean and SEM (**P < 0.01;*P < 0.05; ns: no significant difference).

We injected mice with OVA-induced asthma intraperitoneally with PKH26-labeled DCim or DClps or with saline-diluted PKH26 as a control. We screened DCs by FACS with gating on CD11c + CD80 + MHCII + cells in the mononuclear cell populations of lung digests. PKH26-labeled BMDCs were also detected by FACS. On the first day after transplantation, 2.26% of DClps in the lungs were PKH26 positive. The proportion of PKH26-positive BMDCs in the DClps group peaked at 5.42% on day 3; this proportion was significantly higher than that in the DCim (1.52%) and control groups (0.86%). The number of PKH26-positive BMDCs in the DClps group was reduced on day 7 but was still higher than the numbers in the DCim and control groups (Fig. 6). These data showed that DCs delivered intraperitoneally accumulated in the lungs of OVA-sensitized asthmatic mice during the week after passive transfer. The efficiency of DC pulmonary migration was related to the maturation state of DCs, indicating that DClps could reach the lungs in more meaningful numbers than DCim.

### Administration of DClps in an OVA-induced mouse model significantly alleviated allergic airway inflammation

After we confirmed that the efficiency of pulmonary migration was determined by the maturation state of DCs, we attempted to further clarify whether the degree of DC maturation affected histopathological changes in the lungs.

**Figure 7:**
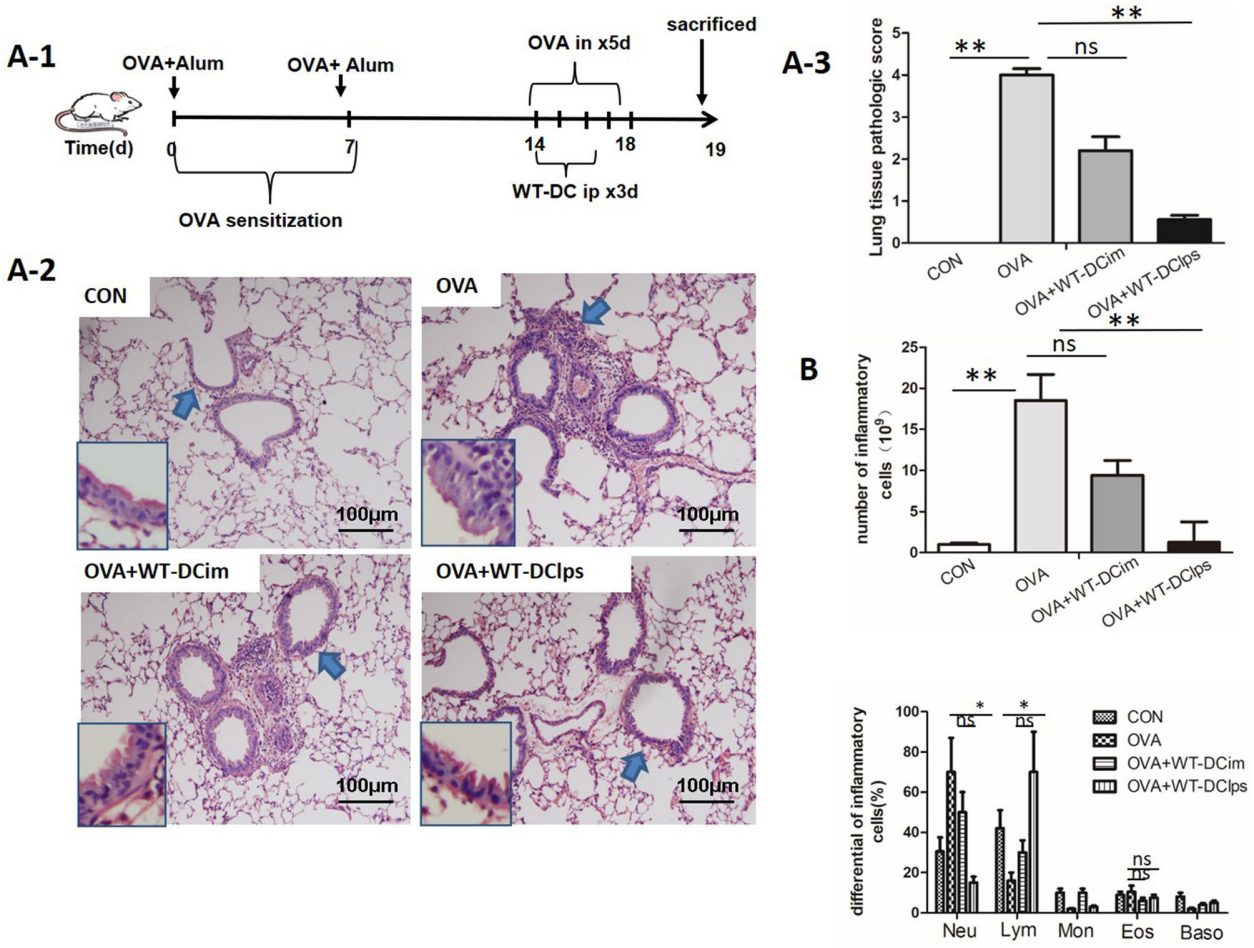
Effect of adoptively transferring LPS-pulsed mature BMDCs on lung pathology in OVA-induced asthmatic mice. (**A-1**) Protocol for OVA-mediated induction of allergic asthma and transperitoneal adoptive transfer of LPS-pulsed matured BMDCs (wild type) into OVA-sensitized mice. (**A-2**) Representative photomicrographs of lung sections stained with H&E and examined at × 100 magnification. The same experiment was repeated 3 times with similar results (n = 6 in each group). (**A-3**) The scores for lung tissue pathology. (**B**) The total cell number in the bronchoalveolar lavage fluid (BALF) (top panel). The differential cell counts in the BALF (bottom panel). The columns and error bars represent the mean and SEM (**P < 0.01;*P < 0.05; ns: no significant difference).

In vivo experiments revealed that compared with DCim injection or no DC injection, DClps injection significantly reduced lung inflammatory cell infiltration and lung tissue pathological scores in mice with OVA-induced asthma (Fig. 7A). In addition, DClps injection significantly reduced the total number of pulmonary inflammatory cells in BALF, while there was no statistically significant difference between the DCim + OVA and OVA groups (Fig. 7B). Moreover, neutrophil infiltration was reduced and lymphocyte counts were increased in both the DClps + OVA and DCim + OVA groups (Fig. 7B).

### Administration of DClps reversed impairments in the STAT4 and STAT6 pathways

**Figure 8:**
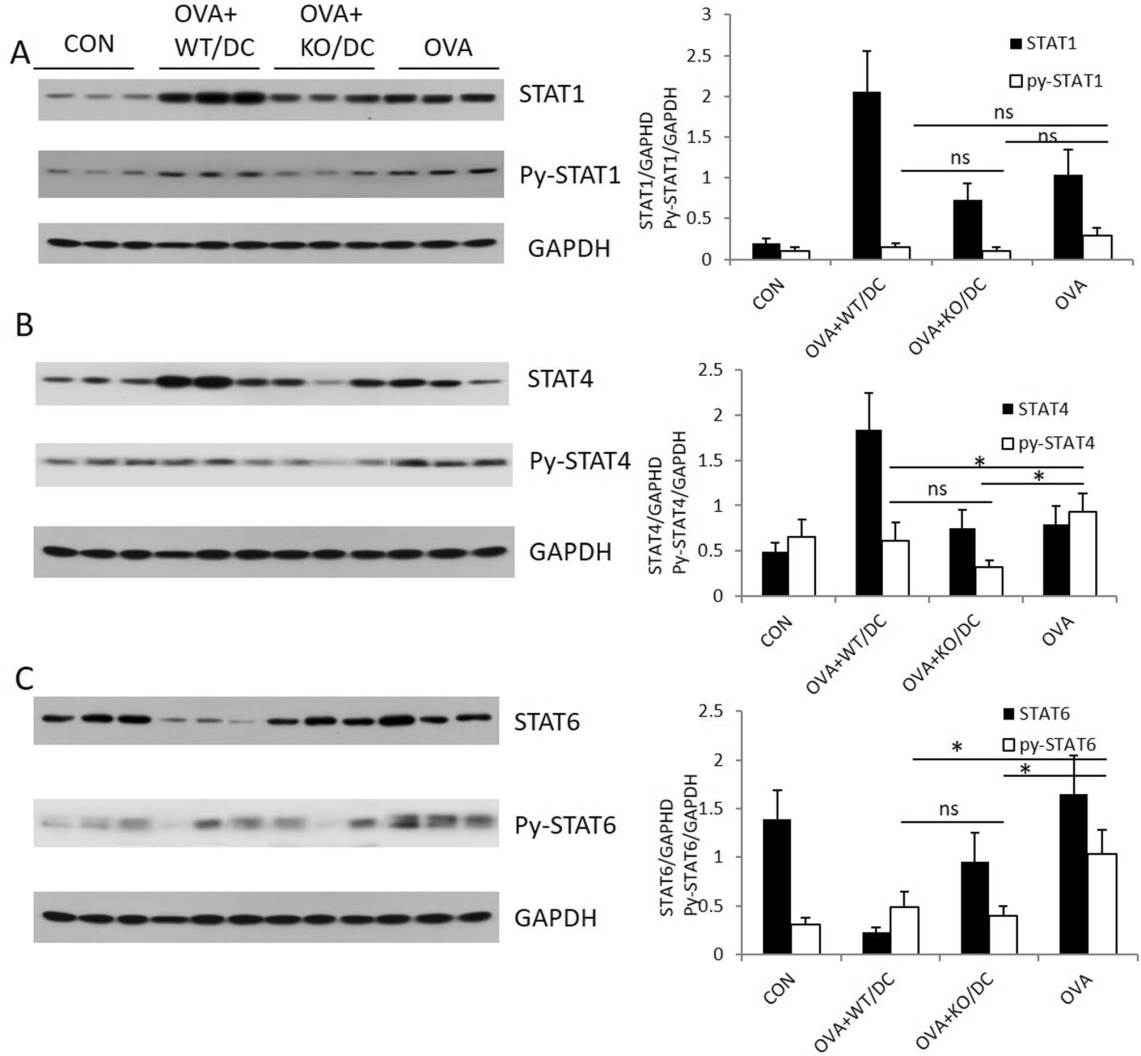
STAT signaling pathways were inhibited by adoptive transfer of BMDCs with or without the SOCS3 gene. Lung tissue samples were homogenized after BMDC adoptive transfer into OVA-sensitized mice. Mononuclear cells were isolated from homogenates. STATs and phosphorylated STATs were detected by Western blotting (WB). The right columns represent the densitometry analysis of the WB results. (**A**) STAT1 and py-STAT1 detected by WB. (**B**) STAT4 and py-STAT4 detected by WB. C. STAT6 and py-STAT6 detected by WB. Data are representative of 3 independent experiments with similar results (*P < 0.05, ns: no significant difference).

To define the roles of DCs in the Th1/Th2 immune signaling pathways, we evaluated the activation and expression of STAT-1, STAT-4, and STAT-6 in different groups of mice (the control [CON], OVA + WT DC [WT-DC], OVA + KO DC [KO-DC], and OVA groups). Western blot analysis revealed that administration of DCs significantly reduced the phosphorylation of STAT-4 and STAT-6 in the lungs of OVA-sensitized mice, and this effect was not related to the lack of SOCS3 (Fig. 8). There was also a trend toward reduced STAT1 phosphorylation after administration of DCs in mice with OVA-induced asthma. However, the reduction was not significant (Fig. 8). These findings indicate that DCs have the capacity to inhibit STAT6 and STAT4 signaling pathways to regulate allergen-induced Th2 immune responses.

## Discussion

The role of the SOCS3 gene in inflammatory diseases, such as allergic asthma, has not been well defined. The SOCS3 protein is the most widely studied member of the SOCS family, which includes negative regulators of cytokine signaling. SOCS3 is central in negatively regulating Janus kinase (JAK) and STATs, such as STAT3, STAT4, STAT1 and STAT5. Previously published evidence has shown that SOCS3 knockdown leads to improvements in inflammation and airway hyperreactivity (AHR) in asthmatic mice and that T cell or CD4+ T cell activation is restricted after specific SOCS3 depletion^11^. However, Duan et al. found that deletion of SOCS3 in macrophages enhanced the expression of STAT1-stimulated genes in response to IL-6. In addition, systemic administration of a SOCS3-specific antagonistic peptide (pJAK2) resulted in the induction of IFN responses^12^. Our previously published data revealed that SOCS3-deficient bone marrow-derived macrophages (BMDMs) expressed relatively high levels of TNF-α and that adoptive transfer of SOCS3-deficient BMDMs into WT mice enhanced the severity of acute lung injury (ALI)^10^. Such inconsistencies might be related to the different cell subsets in which the SOCS3 gene was specifically depleted in our experiments (myeloid cells) and other experiments (T cells). Based on previous data, BMDCs with or without SOCS3 gene expression were pulsed with LPS in the current study to promote maturation. We found that either SOCS3^−/−^ DC transfer or SOCS3^+/+^ DC transfer markedly alleviated OVA-induced lung injury and dramatically decreased the total number of inflammatory cells in BALF. When we examined the proportions of cells in BALF, we found that a lower proportion of neutrophils and a higher proportion of lymphocytes were present after BMDC adoptive transfer. Th2-type cytokines and IgE levels were also markedly decreased after BMDC transfer. Interestingly, compared with WT DCs, SOCS3^−/−^ DCs slightly attenuated BMDC-induced immunogenic tolerance. In this regard, the effect of SOCS3 depletion on BMDC function was evaluated in an MLR. After mixing cultured BMDCs with CD4+ T cells, we found that the T cell proliferative capacity was enhanced in the SOCS3^−/−^ BMDC group compared with the SOCS3^+/+^BMDC group and the control group. SOCS3^−/−^ BMDCs induced CD4 + T cells to produce more IFN-γ but not IL-4. This finding partially explains why BMDC-induced immunogenic tolerance was slightly attenuated by SOCS3^−/−^ BMDCs compared with SOCS3^+/+^BMDCs.

Different DC subsets and their discrete functional states might be responsible for promoting immunologic tolerance rather than inflammation. pDCs constitute a unique DC subset with the ability to induce regulatory T (Treg) cell responses and inhibit Th2 cell production, which has the potential to induce antigen-specific tolerance in asthma^13,14^. Kool et al.^15^ previously reported that selective removal of pDCs during allergen stimulation enhances airway inflammation, while adoptive transfer of pDCs before allergen stimulation inhibits airway inflammation. In contrast, adoptive transfer of conventional DCs (cDCs) or monocyte-derived DCs (moDCs) into OVA-sensitized mice augments airway inflammation. The various functional states of DCs affect allergic airway inflammation and AHR differently. Previous findings have shown that airway delivery of OVA-pulsed splenic CD8α + DCs reverses AHR and Th2 responses but not allergen-specific IgE and IgG1 responses^16^. In addition, adoptive transfer of TGF-β and IL-10-treated DCs can significantly attenuated asthma presentation in sensitized mice^17^. On the other hand, DCs differentiated with GM-CSF enhance AHR and eosinophil numbers and augment Th2 responses in recipient mice. Similar results have been observed in mice transferred with TNF-treated DCs^14^. In this study, a subset of BMDCs were pulsed with LPS for 24 h to induce DClps development, while another subset of BMDCs that were not pulsed with LPS were called DCim. We found that adoptive transfer of DClps, but not DCim, via intraperitoneal injection greatly improved lung pathology scores and alleviated airway inflammatory cell infiltration. We subsequently evaluated the biological activities of DClps and DCim. DClps secreted more IL-10 and TGF-β than DCim, regardless of whether the SOCS3 gene was expressed. This finding is consistent with previous data showing that IL-10 plays a very important role in IL-10-differentiated DC (DC10)-mediated AHR improvement in allergic mice. The levels of MCP-3 and IFN-γ production were not significantly changed by BMDCs.

Gordon et al.^3^ assessed the effectiveness of DC10 delivery to asthmatic animals via various routes, specifically the transtracheal (t.t.), intraperitoneal (i.p.), intravenous (i.v.) and subcutaneous (s.c.) routes, and found that t.t. DC10 delivery and i.p. DC10 delivery were equally effective in reversing AHR and rapidly inhibiting eosinophil infiltration and the Th2 response. S.c. DC10 transfer inhibited airway Th2 responses to allergen attack, and i.v. DC10 transfer was completely ineffective in inducing tolerance in asthmatic mice^6^. In the present study, PKH26-labeled BMDCs were transferred into OVA-sensitized mice via i.p. injection. We found that DClps could migrate to OVA-sensitized lungs more effectively than DCim. DClps began to migrate to the mouse lungs one day after delivery, and their numbers peaked on day 3 (5.42% vs 1.52% for DClps vs DCim; 5.42% vs 0.86% for DClps vs saline control). The labeled BMDCs in both groups disappeared on day 7. We found that migrated BMDCs prevented OVA-sensitized mice from reestablishing Th2 inflammation when BMDCs were administered intraperitoneally on days 14–16 during the OVA challenge period. This result indicates the rapid effects of BMDCs on immunological tolerance after adoptive cell transfer. However, the published data regarding the time frames of DC tolerance are inconsistent. For example, Nayyar et al.^18^ reported that the attenuation of AHR was first apparent 2 weeks after DC10 delivery and that the effect lasted for 3–10 weeks. We note that there are differences in experimental design, however; Nayyar et al. used mice with chronic OVA-induced asthma and performed DC10 transfer 2 weeks after OVA challenge, which was quite different from our approach.

The tolerogenicity of DCs is controlled by a complex network of environmental signals and intrinsic cellular mechanisms. DCs interact with T cells and determine the differentiation of distinct T cell subsets, such as the Th1, Th2, and Treg cell subsets. Th1 cells are primed mainly through the IFN-γ/STAT1 pathway and the IL-12/STAT4 pathway, while Th2 cells are primed mainly through the IL-4/STAT6 pathway. Medoff et al.^19^ demonstrated that STAT6 in BMDCs was sufficient for the production of C–C motif chemokine ligand (CCL) 17, CCL22, and CCL11, which are critical for Th2 lymphocyte recruitment to allergic airways, and found that STAT6 deficiency abrogated Th2 cell-selective chemokine production in an asthmatic mouse model. In the present study, we found that the phosphorylation of STAT4 and STAT6 in lung mononuclear cells was significantly decreased after BMDC adoptive transfer. There was also a decreasing trend in the phosphorylation of STAT1 after BMDC adoptive transfer. However, the difference was not significant. These results indicated that i.p. BMDC adoptive transfer reversed OVA-sensitized airway inflammation by inhibiting proinflammatory signal transcription and ultimately depressing both Th1- and Th2-mediated inflammation, especially Th2-mediated inflammation.

Overall, the findings suggested that BMDC adoptive transfer-induced immunogenic tolerance in OVA-sensitized mice might not be due to SOCS3 gene depletion. SOCS3^−/−^ DCs slightly attenuated BMDC-induced immunogenic tolerance. In addition, DClps produced more IL-10 and TGF-β than DCim, leading to dramatic decreases in the phosphorylation of both STAT4 and STAT6, which are critical in initiating Th1- and Th2-mediated immunoinflammatory processes in asthma, respectively. Our study indicates that BMDC adoptive transfer may be developed into a new approach for the treatment of asthma, as it enables fine modulation of the balance between immune tolerance and inflammation. Further exploration is needed to elucidate the promising roles of BMDCs in alleviating allergic diseases. For example, the effects of distinct BMDC subsets on biological functional states, BMDC-T cell interactions, BMDC-epithelium interactions, and relevant molecular and biochemical mechanisms after BMDC transfer warrant additional investigation.

## Methods

### Establishment and identification of myeloid cell-restricted SOCS3-KO mice

The animal protocol for this study was approved by the Ethics Committee of Zhongshan Hospital of Fudan University. All animals used in this study were maintained under specific pathogen-free conditions and treated in accordance with the National Institutes of Health Guide for the Care and Use of Laboratory Animals (NIH Publication No. 8023, revised 1978). The study was approved by the Ethics Boards of Zhongshan Hospital of Fudan University (Approval No. B2014-108).

The generation of SOCS3-KO mice has been described previously^1^. Briefly, conditional SOCS3(Lyz2cre) mice were established by serial breeding of SOCS3fl/fl mice with Lyz2-Cre transgenic mice with Cre under the control of the myeloid cell-restricted Lyz2 promoter (Fig. 1A). Exon 2 was deleted by the Cre protein in SOCS3 Floxp+/+/Lyz2Cre+/− or SOCS3 Floxp+/+/Lyz2Cre+/+mice. Successful deletion of SOCS3 in SOCS3(Lyz2cre) mice was confirmed by PCR methods using 4 pairs of primers according to our previously published methods^1^ (Fig. 1B). The Cre + loci were identified by 700-bp bands, and exon 2-deleted SOCS3-null loci were identified by 250-bp bands (Fig. 1B). The mice identified as having the Cre+/+SOCS3^−/−^ genotype were considered SOCS3(Lyz2cre) mice with myeloid cell-specific deletion of the SOCS3 gene.

To identify SOCS3 expression in BMDCs, bone marrow cells were collected from the femurs and tibiae of WT and SOCS3(Lyz2cre) mice. The bone marrow cells were seeded in RPMI-1640 medium supplemented with 1% antibiotics/antimycotics and 10% heat-inactivated fetal calf serum (FCS) containing 20 ng/ml GM-CSF. On days 3, 6, and 8, the nonadherent (dendritic) cell suspension was collected, and half of the supernatant was left. Complete medium supplemented with 20 ng/ml GM-CSF was added for further culture. On day 9, cells were collected for further study. The expression of cluster of differentiation (CD) 11c, CD80 and MHC-II was used to phenotypically identify BMDCs. A total of 10^6^ cells were washed and subsequently stained with APC-Cy7-conjugated anti-CD11c (clone HL3, BD Pharmingen, USA), PE-Cy7-conjugated anti-CD80 (clone 16-10A1, BD Pharmingen, USA), and FITC-conjugated anti-MHC-II (clone M5/114, BD Pharmingen, USA) antibodies. The cells were stained with an anti-mouse SOCS3 antibody (ab16030, Abcam, UK) after being treated with a fixation/permeabilization agent (554722, BD Biosciences) to assess the SOCS3 expression deficiency in SOCS3-KO mice. The cells were analyzed on a FACSCanto II instrument (BD Biosciences, San Jose, CA, USA), and the data were analyzed with FlowJo software.

### Generation of an OVA-induced mouse asthma model and adoptive transfer of DCs into asthmatic mice

To establish an asthmatic mouse model, mice were sensitized with two i.p. injections of 100 µg of OVA/alum (Sigma-Aldrich, Grade V; St. Louis, MO, USA) on day 0 and day 7 and were then exposed for 20 min to 100 μg of intranasal (i.n.) OVA on 5 consecutive days under light isoflurane anesthesia. The animals were treated with 1 × 10^6^ DCs via i.p. injection on days 14–16 (Fig. 3A).

The mice were sacrificed within 24 h after the last OVA challenge, and BAL was immediately performed using 3 × 1 mL of 0.05 mM PBS-EDTA (Calbiochem, Darmstadt, Germany) as previously described^20–22^. The cells in the BALF were collected with a Cytospin centrifuge (1,200 rpm for 10 min at 4 °C) and stained with Wright’s solution for differential cell counting. The supernatants were collected and frozen at -80 °C for IL-5 and IL-13 assessment by ELISA. Serum and tissue samples were obtained for further analyses.

### Cell preparation and culture of BMDCs

BMDCs were generated from bone marrow cells collected from WT C57/B6 mice and SOCS3-KO mice. The bone marrow cells were collected from the femurs and tibiae of WT and SOCS3(Lyz2cre) mice. The bone marrow cells were then seeded in RPMI-1640 medium supplemented with 1% antibiotics/antimycotics and 10% heat-inactivated FCS containing 20 ng/ml GM-CSF. On days 3, 6, and 8, the nonadherent (dendritic) cell suspension was collected, and half of the supernatant was left. Complete medium supplemented with 20 ng/ml GM-CSF was added for further culture. On day 9, a subset of cells were collected for further study. Another subset of cells were incubated with LPS (100 ng/ml) for 24 h to generate DClps. The cells were then pulsed with OVA (1 µM) for 2 h at 37 °C.

### T cell purification and DC stimulation of T cells

To establish an MLR, CD4 + T cells were first obtained from the spleen and lymph nodes of a native C57/B6 mouse. BMDCs were isolated and cultured as described above. SOCS3^+/+^ and SOCS3^−/−^ BMDCs were pretreated for 20 min at 37 °C in culture medium containing 200 μg/ml mitomycin C (Kyowa Hakko Kogyo, Tokyo, Japan). Murine CD4 + T cells were separated by negative isolation using a MagniSort™ Mouse CD4 T Cell Enrichment Kit (Thermo Fisher). Prior to culture, the purified CD4 + T cells were labeled with CFSE (Invitrogen, Ltd., UK) for subsequent assessment of T cell proliferation. Mitomycin C-treated SOCS3^+/+^ or SOCS3^−/−^ DCs were cocultured with CD4 + T cells at a ratio of 1:4 in a 37 °C, 5% CO2 atmosphere for 5 days. T cell proliferation was assessed by evaluating CFSE dilution on the last day of culture. After stimulation with phorbol 12-myristate 13-acetate (PMA; 100 ng/ml, Sigma) and ionomycin (5 μmol/L, Sigma) overnight, the supernatants of the cocultured cells were collected, and IL-4 and IFN-γ production was determined by ELISA.

### Tracking of SOCS3^−/−^ BMDCs in the lungs

SOCS3^+/+^ and SOCS3^−/−^ BMDCs were labeled with the fluorescent dye PKH26 (mini126, Sigma‐Aldrich) according to the manufacturer's protocol. Briefly, 2 × 10^6^ BMDCs were suspended in 1 ml of diluted buffer from the manufacturer's labeling kit. The cell suspension was mixed with an equal volume of a labeling solution containing 4 × 10^–6^ mol/L PKH26 dye in dilution buffer, and the mixture was incubated for 4 min at room temperature. The reaction was terminated by adding 2 ml of fetal bovine serum (FBS). After washing with a control medium, 5 × 10^6^ DCs labeled with PKH26 were mixed with 1 mol/L PLA‐CMC solution and intraperitoneally injected into mice with OVA-induced asthma.

PKH26-labeled SOCS3^+/+^ and SOCS3^−/−^ DCs were injected intraperitoneally into asthmatic mice (1 × 10^6^ cells/mouse) as described above. After 1, 3 or 7 days, we collected the lung tissue from each animal and generated single-cell suspensions by enzymatic digestion of the lungs (which included cutting the lungs into small pieces, incubating them with collagenase IV and DNase for one hour and filtering the resultant suspension through a 300-µm mesh filter). PKH26-labeled BMDCs in lung tissue samples were evaluated by assessing the expression of CD11c, CD80, MHC-II and PKH26 with a FACSCanto II instrument (BD Biosciences, San Jose, CA, USA).

### Histochemistry and assessment of pathological lung injury

After the BALF was collected, the lungs were infused with 4% paraformaldehyde. Then, the trachea was clamped, and the lungs were excised. The left lung was embedded in paraffin. Five-micrometer paraffin sections were obtained and stained with hematoxylin and eosin (H&E). The right lung was collected and frozen at − 80 °C for protein assessment by Western blotting.

To quantify the degree of lung damage, H&E-stained slides were graded in a blinded fashion using a scoring system described previously^23^. A score of 0 indicated that no detectable inflammation was present, a score of 1 indicated that occasional inflammatory cells were present, a score of 2 indicated that most bronchi or vessels were surrounded by a thin layer (one to five cells thick) of inflammatory cells, and a score of 3 indicated that most bronchi or vessels were surrounded by a thick layer (more than five cells thick) of inflammatory cells. Total lung inflammation was defined as the average of the peribronchial and perivascular inflammation scores. All slides were examined by two independent pathologists.

#### ELISA

Cell culture supernatants and BALF samples were evaluated by using commercially available ELISA kits according to the manufacturers’ instructions. IL-4, IL-5, IL-10, IL-13, MCP-3, TGF-β, IFN-γ and IgE in supernatants were detected with ELISA kits (R&D Systems).

### Western blot analysis

The expression levels of STAT1, STAT4, STAT6 and the corresponding phosphorylated proteins in lung digests were analyzed by Western blot analysis. Cell lysates (40 μg) were separated by 10% sodium dodecyl sulfate-PAGE and transferred to polyvinylidene fluoride (PVDF) membranes (Roche, USA). After incubation in a blocking buffer containing 5% skim milk in TBST (12.5 mM Tris–HCl pH 7.5, 68.5 mM NaCl, and 0.1% Tween 20) for 1 h, the blots were incubated overnight with primary antibodies including rabbit anti-STAT1 (D1K9Y, Cell Signaling Technology, USA), rabbit anti-phosphorylated STAT1 (Tyr701, Cell Signaling Technology, USA), rabbit anti-STAT4 (C46B10, Cell Signaling Technology, USA), rabbit anti-phosphorylated STAT4 (ab28815, Abcam, UK), rabbit anti-STAT6 (ab32520, Abcam, UK), and rabbit anti-phosphorylated STAT6 (ab28829, Abcam, UK). An anti-mouse GAPDH antibody was used as a loading control. The blots were washed with TBST, incubated with horseradish peroxidase (HRP)-conjugated anti-rabbit Ig (Jackson ImmunoResearch) and then developed with an enhanced chemiluminescence (ECL) substrate solution (Millipore).

### Statistical analysis

All data analysis and graph preparation were performed with GraphPad Prism 5.01 (GraphPad Software, San Diego, CA, USA). The data are presented as the mean ± standard error for each experimental group. Multigroup comparisons were performed by either one-way ANOVA with Tukey’s post hoc test or Wilcoxon signed rank tests (FACS analyses). P < 0.05 was regarded to indicate significance.

## Supplementary information

Supplementary file1
