# Supplementary material for: Adoptive transfer of bone marrow-derived dendritic cells (BMDCs) alleviates OVA-induced allergic airway inflammation in asthmatic mice
Source: Sci Rep. 2020 Aug 17;10:13915. doi: 10.1038/s41598-020-70467-3 (PMC7431555; doi:10.1038/s41598-020-70467-3)
Supplement: Supplementary file 1 — Supplementary file1 [file 41598_2020_70467_MOESM1_ESM.pdf]

Adoptive transfer of bone marrow-derived dendritic cells (BMDCs) alleviates OVA-induced allergic airway inflammation in asthmatic mice

Kan Xu[#1], Nan Wu[#1], Zhihui Min[#3], Zheng Li[3], Tao Zhu[4,5], Chunfang Liu[6], Yuzhen Zeng[2], Juan Song[2], Ruolin Mao[2], Hong Ji[*5], Zhilong Jiang[*2], Zhihong Chen[*2]

1. Geriatric Department of Zhongshan Hospital, Shanghai Institute of Respiratory Disease, Fudan University, Shanghai, China
2. Respiratory Division of Zhongshan Hospital, Shanghai Institute of Respiratory Disease, Fudan University, Shanghai, China
3. Research Center of Zhongshan Hospital, Fudan University, Shanghai, China
4. Department of Respiratory Medicine, Second Affiliated Hospital of Chongqing Medical University, Chongqing, China
5. Department of Anatomy, Physiology and Cell Biology, School of Veterinary Medicine, University of California, Davis, CA, USA; California National Primate Research Center, Davis, CA, USA
6. Department of Laboratory Medicine, Huashan Hospital, Shanghai Medical College, Fudan University, Shanghai, China

*Correspondence:

Zhihong Chen, MD, PhD. Respiratory Division of Zhongshan Hospital, Shanghai Institute of Respiratory Diseases, Fudan University, No. 180 Fenglin Road, Shanghai, China. Tel: 86-021-64041990-2445. Fax: 86-021-64187165. E-mail: czh60@hotmail.com

Zhiling Jiang MD, PhD. Respiratory Division of Zhongshan Hospital, Shanghai Institute of Respiratory Diseases, Fudan University, No. 180 Fenglin Road, Shanghai, China. E-mail: Jiang.zhilong@zs-hospital.sh.cn

Hong Ji, PhD. Department of Anatomy, Physiology and Cell Biology, School of Veterinary Medicine, University of California; California National Primate Research Center, Davis, CA, USA. Phone: 530-754-0679. E-mail: hgji@ucdavis.edu

# Kan Xu, Nan Wu and Zhihui Min contributed equally to this paper.

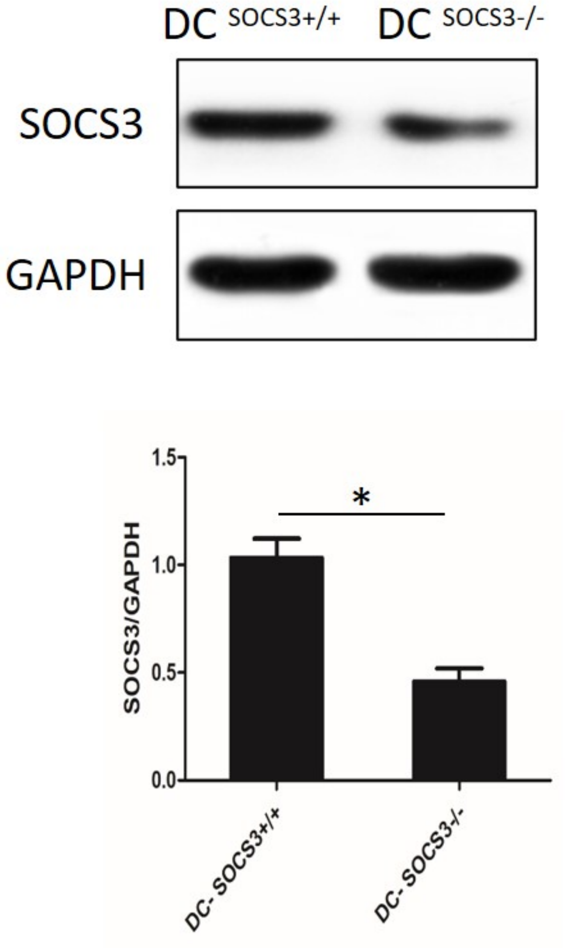
DC SOCS3+/+
DC SOCS3-/-
SOCS3
GAPDH
1.5
1.0
0.5
0.0
SOCS3/GAPDH
*
DC- SOCS3+/+
DC- SOCS3-/-

Supplementary data 1: The protein expression of SOCS3 in SOCS3-/- BMDCs measured by Western blotting (WB).

The experiment was repeated three times, and representative data
showed similar results. The lower columns represent the densitometry analysis of the WB results. （*： $P < 0.05$）
